# Supplementary material for: Sorting at embryonic boundaries requires high heterotypic interfacial tension
Source: Nat Commun. 2017 Jul 31;8:157. doi: 10.1038/s41467-017-00146-x (PMC5537356; doi:10.1038/s41467-017-00146-x)
Supplement: Supplementary file 2 — Supplementary Software 1 [file 41467_2017_146_MOESM2_ESM.zip › PottsModel/SrcPottsModel/doc/index-files/index-12.html]

L-Index


JavaScript is disabled on your browser.


Skip navigation links


- Overview
- Package
- Class
- Use
- Tree
- Deprecated
- Index
- Help

- Prev Letter
- Next Letter

- Frames
- No Frames

- All Classes

A B C D E F G H I J K L M N O P R S T U V W 


## L

latentTime - Variable in class model.Constants.ParameterManager


Lattice - Class in model


Lattice(Constants) - Constructor for class model.Lattice


Lattice(Constants, int[][][]) - Constructor for class model.Lattice
:   Initialize grid with specified pixel IDs.

lattice(Lattice.Type, int, int, int, int, int, int) - Static method in class model.LatticeUtilities
:   Returns lattice of cell IDs and Types of dimension (2 \* width \* height)
    where lattice[0][i][j] returns the cell ID of pixel at position (i,j) and
    lattice[1][i][j] returns the cell type of pixel at position (i,j)

Lattice.PixelIterator - Class in model


Lattice.State - Enum in model


Lattice.Type - Enum in model


LatticeUtilities - Class in model


LatticeUtilities() - Constructor for class model.LatticeUtilities


locateClickedPixel(Point) - Method in class gui.PottsCanvas
:   Return pixel clicked.

A B C D E F G H I J K L M N O P R S T U V W

Skip navigation links


- Overview
- Package
- Class
- Use
- Tree
- Deprecated
- Index
- Help

- Prev Letter
- Next Letter

- Frames
- No Frames

- All Classes
